# The pharmacological void: death narcissism in post-conflict Northern Ireland

**DOI:** 10.1192/bjb.2025.10207

**Published:** 2026-04

**Authors:** Adam Flynn

**Affiliations:** M.B., MRCPsych, School of Psychiatry, Northern Ireland Medical & Dental Training Agency, Belfast, UK

Northern Ireland continues to record some of the highest drug-related death rates in the UK, second only to those in Scotland.^1^ Yet, the toxicology reveals a distinct narrative. Unlike trends driven elsewhere by illicit ‘street’ markets, Northern Ireland’s crisis is disproportionately fuelled by misuse of prescribed sedatives. The prevalence of gabapentinoids (specifically pregabalin), benzodiazepines and opioids suggests that the goal of the user is not to enhance reality but to erase it. Indeed, the rates of diazepam prescription alone are 3.5 times higher per capita than in England.^2^

To understand this, we must look beyond simple socioeconomic determinants and turn to the psychoanalytic framework of French theorist André Green. Green distinguished between life narcissism, the drive for unity and connection, and death narcissism, the drive to reduce psychic tension to absolute zero.^3^

Standard addiction models often focus on the pleasure principle (the ‘high’). However, the clinical presentation of the Northern Irish patient often aligns more closely with Green’s *The Work of the Negative*. The user of pregabalin or diazepam is arguably not seeking euphoria but is engaging in a chemical form of ‘negative hallucination.’^4^ They are attempting to create a blank space where perception, memory and trauma are rendered non-existent.

This ‘drive for zero’ resonates deeply with the post-conflict Northern Irish collective psyche. Green’s concept of the ‘dead mother’ describes a caregiver who is physically present but emotionally absent, leaving the child with a ‘psychic hole.’ It is not a leap to suggest that for many, the Peace Process that ended large-scale violence and trauma here has become a societal dead mother, with the removal of the intrusive object (violence) replaced not by a living structure but by an institutional void that our dysfunctional governmental façade encapsulates.

In this context, these specific drugs act as a somatic short-circuit. They allow the individual to ‘un-see’ the emptiness of the post-conflict landscape and ‘un-feel’ the intergenerational trauma that remains unarticulated. The drug is not a recreational object; it is a psychosomatic shield^5^ against what Green called ‘private madness.’

As psychiatrists, we must recognise that when we strip away these medications, we are not just removing a chemical dependency, we are exposing the patient to the ‘white noise’ of their own internal void. Addressing this crisis requires us to treat not just the addiction but the underlying ‘death narcissism’, the profound, often silent desire for non-existence that haunts the post-conflict subject and population.
